# Towards smoke-free cars in the Republic of Korea: Evidence from environmental and biochemical monitoring of third-hand smoke exposure in taxis

**DOI:** 10.18332/tid/85089

**Published:** 2018-03-22

**Authors:** Eun Young Park, Min Kyung Lim, Sun Yeol Hong, Jee Eun Oh, Bo Yoon Jeong, E Hwa Yun, Wonho Yang, Do-Hoon Lee

**Affiliations:** 1 Division of Cancer Prevention, National Cancer Control Institute, National Cancer Center, Goyang, Republic of Korea; 2 Department of Cancer Control and Population Health, Graduate School of Cancer Science and Policy, National Cancer Center, Goyang, Republic of Korea; 3 Department of Laboratory Medicine, Center for Diagnostic Oncology, National Cancer Center, Goyang, Republic of Korea; 4 Division of Health and Nutrition Survey, Korea Centers for Disease Control and Prevention, Ministry of Health and Welfare, Osong, Republic of Korea; 5 Department of Occupational Health, Catholic University of Daegu, Daegu, Republic of Korea

**Keywords:** third-hand smoke, taxi, smoke-free policy, air nicotine, dust nicotine-derived nitrosamine ketone

## Abstract

**INTRODUCTION:**

To evaluate the level of tobacco smoke exposure in taxis in Korea using tobacco specific environmental markers.

**METHODS:**

From June to September 2012, cross-sectional measurements of air nicotine levels and dust nicotine-derived nitrosamine ketone (NNK) concentrations were conducted in 17 taxis in Goyang, Korea. Field investigators completed an observational questionnaire on building characteristics, smoking policies and evidence of smoking. Descriptive statistics including geometric means (GMs) ± standard deviations were produced for air nicotine levels and dust NNK concentrations.

**RESULTS:**

There was no evidence of active smoking in the 17 taxis monitored, despite the fact that 10 drivers were current smokers. The overall GMs of air nicotine and dust NNK concentration were 0.42 μg/m^3^ and 6.78 pg/mg, respectively. These levels were 3.4-fold and 2.6-fold higher in taxis whose drivers were current smokers compared to the taxis of nonsmokers (GM of air nicotine: 0.65 μg/m^3^ vs 0.19 μg/m^3^; GM of dust NNK: 10.07 pg/mg vs 3.85 pg/mg).

**CONCLUSIONS:**

The present study shows that air nicotine and dust NNK were detected in all taxis regardless of whether the taxi driver was smoking or not, which indicates the potential for exposure to SHS or THS. It suggests that an appreciable level of SHS and TSH exposure might occur if the environment is not completely smoke-free and enforcement is lacking.

**ABBREVIATIONS:**

THS: third-hand smoke, SHS: second-hand smoke, FCTC: Framework Convention Alliance for Tobacco Control, NNK: 4-(methylnitrosamino)-1-(3-pyridyl)-1-butanone, LOD: limit of detection, GM: geometric mean, GSD: geometric standard deviation.

## INTRODUCTION

Second-hand smoke (SHS) has been established as toxic matter; no level of SHS exposure is safe. It causes among non-smoking adults preventable adverse health outcomes, such as cardiovascular diseases, cancers and premature deaths, and likely associated with higher risks of middle-ear disease, impaired lung function, sudden infant death syndrome, lower respiratory illnesses, and infections^1^. Therefore, over the previous decade, many countries worldwide have made substantial progress in reducing SHS exposure in most indoor public places, including public transportation vehicles such as public buses, passenger railways, taxis, and other business vehicles^2^.

Motor vehicles, including taxis, are a small and often fully enclosed space (if windows are closed), conditions that result in high SHS concentrations. This may pose a threat to the health of passengers, especially susceptible individuals such as pregnant women, children and the elderly^3^. Levels of total particulate matter and air nicotine have been used as indices of SHS in motor vehicles, and were found to be comparable to or higher than those reported in entertainment venues^4,5^. Short (1 hour) SHS exposure in cars was found to induce considerable increases in the levels of tobacco biomarkers in non-smokers^6^. Furthermore, exposure to SHS in vehicles has been associated with an increased risk of respiratory and allergic symptoms in children and young adults^7,8^.

In this respect, taxis, as well as other public transport vehicles, are included in lists of mandatory smoke-free places in many European countries, some USA States, Canada, Australia etc^9^. In Korea, smoking prevalence decreased from over 70% in the 1990s to 39% in 2015 owing to the ratification and implementation of FCTC articles, including continuous and effective mass media campaigns, recent tax raises, pictorial warnings on cigarette packs, expanding legislated smoke-free places, and diverse national cessation services. However, enforcement of these policies is still lacking^10,11^. Even though other public transport vehicles with a capacity of more than 16 passengers or those used for transporting children were designated as smoke-free places in Korea by the National Health Promotion Law^12^, taxis had not been included in the law and drivers’ smoking was permitted in the absence of passengers. Although taxis have been included in the mandatory smoke-free places by the Passenger Transport Service Act in Korea since 2014^13^, it is not well known to the public. Based on previous study results that suggested poor enforcement of the smoking ban in taxis and that a high smoking prevalence could lead to low compliance^14^, exposure to SHS or third-hand smoke (THS) in taxis was unavoidable in Korea, while research and information on this exposure is lacking.

On the other hand, although various measures are used to assess smoking exposure, levels of nicotine and tobacco-specific nitrosamines, such as 4-(methylnitrosamino)-1-(3-pyridyl)-1-butanone (NNK), in the air and on environmental dust, respectively, are excellent markers of SHS or THS exposure^15^.

Thus, the purpose of the present study was to evaluate the level of tobacco smoke exposure in taxis using the above-mentioned tobacco-specific environmental markers when the smoking in taxis was not completely banned or enforced in Korea.

## METHODS

From June to September 2011, cross-sectional measurements of air nicotine levels and dust NNK concentrations were conducted in taxis in Goyang, a medium-sized and residential satellite city of the capital city, Seoul. We randomly selected a company in Goyang and then randomly selected taxis of the company to measure exposure to tobacco smoke within them, and also conducted a questionnaire survey among the taxi drivers. With the cooperation of the company and agreement by the drivers, 17 taxis were finally included in the study and informed consent was obtained from the taxi drivers (10 smokers and 7 non-smokers).

Field researchers completed an observational questionnaire to document evidence of active smoking (smell of cigarette smoke, presence of cigarette butts, witnessing people smoking, etc.) and a questionnaire regarding participants’ history of smoking and SHS exposure (smoking status, average daily SHS exposure, etc.).

The measurements of air nicotine and dust NNK concentrations were performed in a manner similar to our previous study, and followed the protocol of the Flight Attendant Medical Research Institute (FAMRI) study^16,17^. All nicotine concentrations were measured using passive sampler badges installed for a week in the taxis and hung from the roof above the driver’s seat to prevent loss. The sampling rate was 24 mL/min and the calculated limit of detection was 0.05 μg/m^3^ over the 7-day sampling period. The exposed filter was analyzed by gas chromatography using an instrument equipped with a nitrogen phosphorous detector (7820A; Agilent Technologies, Santa Clara, CA, USA).

Dust NNK concentrations were measured using dust samples collected from three interior ledges of taxis. Filter paper that was soaked in 50% MeOH and left to dry was used to collect the dust samples. After collection, 4 mL of 100 mM ammonium acetate was added to extract samples from the paper. Samples were analyzed using Agilent 1260 rapid resolution liquid chromatograph (Agilent Technologies, Santa Clara, CA, USA) coupled with a triple quadruple mass spectrometer equipped with a Turbo Ion Spray TM source (AB SCIEX, Framingham, MA, USA). Standard curves were made from blank dust created by spiking a blank matrix with 100 mM ammonium acetate. The limits of detection (LOD) and limit of quantification (LOQ) for NNK concentration were 5 pg/mg (S/N 3.2) and 25 pg/mg (S/N 7.2), respectively. The precision (both within-day and between-day) of the method was found to be acceptable (coefficient of variance < 10%).

Descriptive statistics, including geometric means (GMs) ± geometric standard deviations (GSDs), were produced for air nicotine levels and dust NNK concentrations. Statistical analyses were carried out using the SAS software (ver. 9.2; SAS Institute, Inc., Cary, NC, USA).

The present study was approved by the Institutional Review Board of the National Cancer Center, Korea.

## RESULTS

Three air nicotine samplers were lost during the monitoring period. Air nicotine and dust NNK concentrations were below the LOD in three taxis and one taxi, respectively. There was no evidence of active smoking - such as smell of cigarette smoke, presence of cigarette butts, or witnessing people smoking - in any taxi during the monitoring period, despite the fact that 10 of the 17 drivers were current smokers.

The overall GMs of air nicotine and dust NNK concentration were 0.42 μg/m^3^ and 6.78 pg/mg, respectively, in the taxis monitored. These measures were 3.4-fold and 2.6-fold higher in taxis whose drivers were current smokers than for taxis driven by non-smokers (GM of air nicotine: 0.65 μg/m^3^ vs 0.19 μg/m^3^, GM of dust NNK: 10.07 pg/mg vs 3.85 pg/mg, respectively) (Table 1).

**Table 1:** Geometric means ± standard deviations of air nicotine and dust nicotine-derived nitrosamine ketone (NKK) levels inside taxis in the Republic of Korea

Measures	N	Mean	SD	GM	GSD	Min	25th§	Median	75th§	Max
**Total**										
Air nicotine	14*	0.754	0.758	0.422	3.903	0.040	0.531	0.582	0.976	3.048
Dust NNK	17	10.044	9.130	6.777	2.524	2.500	2.500	7.321	13.017	31.027
**Non-smokers**										
Air nicotine	4*	0.351	0.284	0.194	4.235	0.040	0.040	0.531	0.554	0.589
Dust NNK	7	4.793	3.948	3.850	1.936	2.500	2.500	2.500	7.321	12.806
**Current smokers**											
Air nicotine	10	0.978	0.857	0.649	3.247	0.040	0.576	0.656	1.032	3.048
Dust NNK	10	13.719	10.066	10.067	2.460	2.500	7.013	10.693	24.554	31.027

SD: standard deviation, GM: geometric mean, GSD: geometric standard deviation, Min: minimum value, Max: maximum value, NNK: nicotine-derived nitrosamine ketone.

§ percentile

* Three of the seventeen air nicotine passive sampler badges were lost during the sampling period.

## DISCUSSION

To the best of our knowledge, this represents the first field monitoring study providing scientific evidence of potential THS exposure in taxis in the Western Pacific region, where the prevalence of smoking is still high and the population has a social acceptance of smoking.

Although there was no evidence of active smoking, air nicotine and dust NNK were detected in all taxis including non-smokers’ taxis, at appreciable levels, and obviously, were higher in the taxis whose drivers were current smokers. This indicates the potential for exposure to SHS or THS. Meanwhile, air nicotine and dust NNK detected in non-smokers’ taxis was likely to reflect THS, although SHS due to smoking by passengers in taxis was not entirely excluded. In Korea, awareness of the effects of SHS exposure has increased owing to national anti-tobacco campaigns focusing on the necessity for smoke-free policies to avoid SHS exposure and the tabling of laws to increase the number of smoke-free places^18^. In this political and social context, it was not publicly acceptable to smoke inside a taxi, which is a small fully enclosed space. However, there were probably some taxi drivers who smoked in the taxi without passengers or some passengers who smoked in the taxi without concern of the social unacceptability.

According to the International Tobacco Control Policy Evaluation (ITC) Korea National Report, 70% of smokers did not smoke in a car or other private vehicle when accompanied by a non-smoker^19^. In addition, a previous study conducted in Lisbon reported that taxi drivers might smoke in the taxi when they are alone, and smoker taxi drivers might allow the passengers to smoke despite the smoking ban^14^. Although the majority of drivers and passengers who are current smokers usually do not smoke in the taxi, they smoke before entering the taxi, and residual nicotine and other chemicals from tobacco smoke might be introduced by their breath, skin, clothes and other surfaces. Even if drivers attempt to eliminate tobacco smoke by airing, opening windows, and by using fans or air conditioners, THS residue accumulates on surfaces over time and resists normal cleaning procedures. Moreover, car interiors have relatively large surface areas, covered with materials that can absorb pollutants in tobacco smoke, which may lead to the formation of reservoirs of residual tobacco smoke pollutants^3^. Reflecting on these explanations, appreciably increased levels of air nicotine and NNK concentrations were identified in taxis during actual driving in the present study. Furthermore, the NNK and air nicotine concentrations in taxis were similar to, but higher than, the values in most mandatory smoke-free public indoor places - such as government buildings, nurseries and private educational institutions - of our previous study during the same period and using the same methods as in the current study^17^. Evidence of smoking was identified in private educational institutions, but not in other places.

In our study, air nicotine and NNK concentrations were markedly higher in taxis whose drivers were current smokers than in taxis whose drivers were non-smokers. These findings are consistent with the results of previous studies conducted in the USA and Canada. Regardless of a smoking ban, the dust nicotine level in cars of smokers was three-fold (with a ban on smoking in cars) or five-fold (without a car smoking ban) higher compared to the level in cars of non-smokers^20^. Other previous studies reported that smoking only a single cigarette in a car can result in a PM2.5 reaching unhealthy levels; moreover, median air nicotine concentrations in smokers’ vehicles were 9.6 mg/m^3^ (5.3–25.5), but were non-detectable in non-smokers’ vehicles^4,5^.

As mentioned above, exposure to SHS or THS in motor vehicles might be linked to severe harm to the health of non-smokers, particularly in vulnerable populations, regardless of whether a ban on smoking in motor vehicles has been implemented. In some USA States, most EU countries, Australia, etc., motor vehicles, including taxis, are included in the list of legally mandated smoke-free places. Furthermore, in Australia and Canada, legislation banned smoking in all vehicles with passengers under the age of 16 years, which was altered to under the age of 12 years in July 2015. In England, smoking in cars carrying children under 18 years of age was to become illegal from 1 October 2015^21^. However, a cross-sectional study in New Zealand reported a 3.2% prevalence of smoking in cars, and a higher prevalence in areas with lower socioeconomic status. Spain (5.5%) and Italy (6.95%) reported a higher prevalence of smoking in cars compared to New Zealand^22^. In Wales, 9% of children aged 10–11 years responded that they were exposed to SHS in their family vehicle^23^. The British Lung Foundation has estimated that around 430 000 children are exposed to SHS in their family car every week^21^. Consequently, to prevent SHS and TSH exposure, a completely smoke-free environment is required, rather than specifically designated smoke-free places by law.

Even though smoking in a taxi was prohibited in Korea by law in 2014, and more public places were included in the legislated smoke-free places, SHS and TSH exposure in public places, including taxis, continues and is still high (52%) in public places^10^. Therefore, based on the evidence from further studies in Korea, including investigation of the compliance to the ban on smoking in a taxi, the smoke-free implementation policy should be enforced to make smoking in public places less acceptable, so that harm from SHS and TSH is minimized.

### Strengths and limitations

The single measurements, cross-sectional design, small number of taxis included, and lack of information on smoking behaviors of taxi drivers represent the limitations of this study. Therefore, further research with larger samples is necessary to confirm our findings.

## CONCLUSIONS

In the present study air nicotine and dust NNK were detected in all taxis regardless of whether the taxi driver was smoking or not, which indicates a potential for exposure to SHS or THS. Our study suggests that an appreciable level of SHS and TSH exposure might occur if the environment is not completely smoke-free and enforcement is lacking.
